# Interleukin and Growth Factor Levels in Subretinal Fluid in Rhegmatogenous Retinal Detachment: A Case-Control Study

**DOI:** 10.1371/journal.pone.0019141

**Published:** 2011-04-27

**Authors:** Lukas J. A. G. Ricker, Aize Kijlstra, Alfons G. H. Kessels, Wilco de Jager, Albert T. A. Liem, Fred Hendrikse, Ellen C. La Heij

**Affiliations:** 1 Eye Research Institute Maastricht, Department of Ophthalmology, University Hospital Maastricht, Maastricht, The Netherlands; 2 European Graduate School for Neuroscience (EURON), Maastricht, The Netherlands; 3 Department of Clinical Epidemiology and Medical Technology Assessment, University Hospital Maastricht, Maastricht, The Netherlands; 4 Center for Molecular and Cellular Intervention (CMCI), Department of Pediatric Immunology, University Medical Center Utrecht, Utrecht, The Netherlands; 5 Department of Ophthalmology, University Medical Center Utrecht, Utrecht, The Netherlands; La Jolla Institute of Allergy and Immunology, United States of America

## Abstract

**Background:**

Rhegmatogenous retinal detachment (RRD) is a major cause of visual loss in developed countries. Proliferative vitreoretinopathy (PVR), an eye-sight threatening complication of RRD surgery, resembles a wound-healing process with inflammation, scar tissue formation, and membrane contraction. This study was performed to determine the possible involvement of a wide range of cytokines in the future development of PVR, and to identify predictors of PVR and visual outcome.

**Methodology:**

A multiplex immunoassay was used for the simultaneous detection of 29 different cytokines in subretinal fluid samples from patients with primary RRD. Of 306 samples that were collected and stored in our BioBank between 2001 and 2008, 21 samples from patients who developed postoperative PVR were compared with 54 age-, sex-, and storage-time–matched RRD control patients who had an uncomplicated postoperative course during the overall follow-up period.

**Findings:**

Levels of IL-1α, IL-2, IL-3, IL-6, VEGF, and ICAM-1 were significantly higher (*P*<0.05) in patients who developed postoperative PVR after reattachment surgery than in patients with an uncomplicated postoperative course, whereas levels of IL-1β, IL-4, IL-5, IL-7, IL-9, IL-10, IL-11, IL-12p70, IL-13, IL-15, IL-17, IL-18, IL-21, IL-22, IL-23, IL-25, IL-33, TNF-α, IFN-γ, IGF-1, bFGF, HGF, and NGF were not (*P*>0.05). Multivariate logistic regression analysis revealed that IL-3 (*P* = 0.001), IL-6 (*P* = 0.047), ICAM-1 (*P* = 0.010), and preoperative visual acuity (*P* = 0.026) were independent predictors of postoperative PVR. Linear regression analysis showed that ICAM-1 (*P* = 0.005) and preoperative logMAR visual acuity (*P* = 0.001) were predictive of final visual outcome after primary RRD repair.

**Conclusions/Significance:**

Our findings indicate that after RRD onset an exaggerated response of certain cytokines may predispose to PVR. Sampling at a time close to the onset of primary RRD may thus provide clues as to which biological events may initiate the development of PVR and, most importantly, may provide a means for therapeutic control.

## Introduction

Rhegmatogenous retinal detachment (RRD) is an ophthalmologic emergency that occurs in approximately 12.6 cases per 100,000 persons per year [1]. Although an increasingly number of retinal detachments is successfully repaired with a single procedure, proliferative vitreoretinopathy (PVR) is still the primary cause of failure of reattachment surgery [1]. This eye-sight threatening condition is characterized by the formation of cellular membranes on both sides of the retina that upon contraction may cause a redetachment with often a permanent drop in visual acuity. The development of these fibrotic membranes is reminiscent of the normal wound-healing response with inflammation, migration and proliferation of resident ocular cells and invading immune cells [2]. In all these biological processes, cytokines function as signalling molecules between these cells in order to bring about the events that ultimately lead to the formation and contraction of PVR membranes [2].

Several studies have been undertaken to unravel the role of these cytokines in the pathogenesis of PVR by determining their expression in PVR vitreous and PVR membranes [2]–[12]. Resident ocular cells including retinal pigment epithelial (RPE) cells and glial cells, and invading immune cells such as macrophages and lymphocytes are all capable of producing and secreting these cytokines [2]. Immunohistochemical studies have demonstrated the presence of fibroblast growth factor (FGF), insulin growth factor (IGF), and vascular endothelial growth factor (VEGF) in PVR membranes [3], [4]. For example, FGF and IGF have been implicated in the migration and proliferation of RPE cells [5], [6], whereas VEGF may play a role in monocyte chemotaxis [7]. Moreover, levels of interleukin (IL)-1, IL-6, interferon (IFN)-γ, and intercellular adhesion molecule (ICAM)-1 have been shown to be elevated in PVR vitreous [8]–[12], suggesting an inflammatory component in PVR pathogenesis. These data also indicate that not a single cytokine but a complex network of cytokines may underlie the development of contractile fibrotic membranes that are characteristic of PVR.

So far, the use of intravitreal pharmacological agents to prevent the occurrence of PVR has shown disappointing results [13], [14]. Although the intraoperative application of daunorubicin demonstrated some effect by reducing the number of reoperations, its use failed to show a significant increase in reattachment rate in patients with preoperative PVR [15]. A better understanding of the early biological alterations after primary RRD that may lead to postoperative PVR is therefore needed to develop new therapeutic strategies. Previous animal studies have demonstrated an increase in gene and protein expression of proinflammatory cytokines hours and days after the creation of experimental retinal detachment when no PVR membranes have formed yet [16], [17]. However, previous studies have mainly focused on cytokines in ocular fluids of patients with established PVR [11], [12], [18]–[21]. Further, only a limited number of cytokines have been determined in each patient in these studies. Since PVR is the resultant of an inflammatory process in which a multitude of cytokines is involved, the measurement of a single cytokine seems rather unsatisfactory. Therefore, examining a whole cytokine profile in patients after primary RRD provides not only clues as to which cytokines may play a role in PVR pathogenesis, it may also indicate which cytokines are most predictive of its development.

In a previous study on early biological alterations after primary RRD, we used recently developed multiplex bead-based immunoassays for the simultaneous detection of several chemokines and their correlation with IL-6 [22]. In the study presented here we report the protein expression of 29 different interleukins and growth factors in subretinal fluid samples from the same population. Our findings indicate that an exaggerated response of certain cytokines occurring after RRD onset may predispose to PVR development.

## Materials and Methods

### Patients

The research followed the tenets of the Declaration of Helsinki and the Medical Ethics Committee of University Hospital Maastricht, which approved all the protocols used in this study. All enrolled patients gave written informed consent before the surgical procedure and after the nature of the study was explained.

Subretinal fluid samples are routinely collected during scleral buckling surgery for primary RRD in our department, except in patients with small (less than one quadrant) or shallow detachments. Between 2001 and 2008, a total of 306 patients underwent subretinal fluid sampling. Upon collection, samples were transferred and stored in the BioBank Maastricht. A thorough medical record study revealed that 45 of these patients developed a redetachment due to PVR later on during the postoperative follow-up period. Of these, 9 samples were excluded due to low sample volume (<75 µL) or contamination with blood, whereas 6 samples were excluded because of late PVR development (more than 2½ months after subretinal fluid collection). Further, we excluded 4 patients with preoperative vitreous hemorrhage, 4 patients with preoperative trauma, and 1 patient with preoperative cryotherapy. Finally, 21 samples from patients who developed a redetachment due to PVR within 2½ months after scleral buckling surgery for primary RRD were included in the study. This disease group, defined as the PVR group, was compared with patients who did not develop a redetachment during the overall follow-up period (at least 3 months), i.e. patients with an uncomplicated follow-up after primary RRD repair. Samples from these patients served as controls and were defined as the RRD group. We compared every single PVR sample with two to three age-, sex-, and storage-time–matched controls, which resulted in 54 control patients with an uncomplicated follow-up following primary retinal detachment surgery.

### Sample Collection

Undiluted subretinal fluid samples were obtained during scleral buckling surgery for primary RRD. Scleral and choroidal vessels were carefully cauterized before the incision. Any macroscopic blood surrounding the incision opening was removed with a cotton tip. Subretinal fluid was collected from the surface of the sclera with a 25-gauge bent needle. All samples were collected in sterile polypropylene tubes, stored at −80°C, and thawed directly before analysis. Sample volumes ranged between 100–700 µL.

### Multiplex Bead-Based Immunoassay

Cytokines were measured at the Luminex Core Facility (Utrecht, The Netherlands) with a multiplex immunoassay (Luminex, Austin, TX, USA) using an in-house-validated panel which incorporates an appropriate Quality Control Program, as previously described [23]. In summary, the antibody-coated microspheres were incubated for 60 minutes with standards or subretinal fluid (50 µL) in 96-well, 1.2-µm filter plates (Millipore, Amsterdam, The Netherlands). Plates were washed (Bio-Plex pro II wash station, Bio-Rad, Hercules, CA, USA), and a cocktail of biotinylated detection antibodies was added for an additional 60 minutes. After repeated washings streptavidin–phycoerythrin was added and incubated for 10 minutes. Next, after 2 additional washes fluorescence intensity was measured. Data collection and analysis of the data from all assays were performed (Bio-Plex system in combination with Bio-Plex Manager software, ver. 4.1; Bio-Rad, Hercules, CA, USA), by using five-parameter curve fitting. The concentrations of the following cytokines were measured: the interleukins IL-1α, IL-1β, IL-2, IL-3, IL-4, IL-5, IL-6, IL-7, IL-9, IL-10, IL-11, IL-12p70, IL-13, IL-15, IL-17, IL-18, IL-21, IL-22, IL-23, IL-25, and IL-33; the proinflammatory cytokines tumor necrosis factor (TNF)-α and IFN-γ; the growth factors IGF-1, basic (b)FGF, VEGF, nerve growth factor (NGF), and hepatocyte growth factor (HGF); and the cell adhesion molecule ICAM-1. The lower limit of detection of the cytokines ranged between 0.1 and 19.5 pg/mL. Concentrations below the detection limit were assigned the lowest value from the respective standard curve. For statistical analysis, concentrations below the detection limit were converted to a value of 0.5 times the lowest value of the calibration curve.

### Clinical Variables

For all patients, demographic variables, potential clinical risk factors for the development of PVR (Table 1) and the following clinical variables were collected: follow-up time, occurrence of a redetachment, postoperative PVR grade, and preoperative and final postoperative best corrected Snellen visual acuity. PVR was graded according to the Classification of Retinal Detachment with PVR [24]. Data were collected as 0 (no PVR), 1 (grade A), 2 (grade B), 3 (grade C), and 4 (grade D). Duration of retinal detachment was defined as the interval between the onset of symptoms and surgery and was estimated according to a precise history of patients' symptoms. Duration of macular detachment was evaluated separately and was defined as the interval between the onset of a sudden drop in visual acuity and reattachment surgery. The refraction of pseudophakic patients was evaluated based on historical data. For statistical analysis, Snellen visual acuity was transformed into logMAR visual acuity.

**Table 1:** Demographics and Potential Clinical Risk Factors for PVR.

Potential Clinical Risk Factor	RRD Group (*n* = 54)	PVR Group (*n* = 21)	Univariate Testing	Stepwise Logistic Regression
Age (years)	61 (43–79)	62 (43–76)	NS	NS
Sex, Female (%)	26	29	NS	NS
Size of retinal detachment (quadrants)	2 (1–3)	2 (1–4)	NS	NS
Total size of retinal defects (optic disc diameters)	2 (0–5.5)	1 (0–4)	NS	NS
Macular detachment (%)	64	86	NS	NS
Preoperative logMAR VA	0.75 (0.05–2.52)	1.77 (0.10–2.52)	*P* = 0.045	*P* = 0.026
Detachment duration (days)	5 (1–75)	6 (1–90)	NS	NS
Preoperative PVR grade	1 (0–3)	2 (0–3)	NS	NS
Diabetes mellitus (%)	11	10	NS	NS
Preoperative myopia >5D (%)	17	24	NS	NS
Pseudophakia (%)	19	33	NS	NS
Aphakia (%)	0	0	NS	NS
Preoperative uveitis (%)	0	0	NS	NS
Preoperative cryotherapy (%)	0	0	NS	NS
Preoperative vitreous hemorrhage (%)	0	0	NS	NS
Preoperative trauma (%)	0	0	NS	NS

Data are expressed as median (range) or in percentages. Abbreviations: RRD = rhegmatogenous retinal detachment; PVR = proliferative vitreoretinopathy; NS = not significant; logMAR = logarithm of minimal angle of resolution; VA = visual acuity; D = diopters.

### Statistical Analysis

Patients who developed a redetachment due to PVR within 2½ months after the scleral buckling procedure (the PVR group) were compared with patients with an uncomplicated follow-up (the RRD group). Further, we investigated whether cytokine concentrations were different in patients who developed early postoperative PVR (within 30 days after reattachment surgery) compared to patients with late postoperative PVR (more than 30 days after reattachment surgery) and whether differences in cytokine expression were related to PVR grade. The nonparametric Mann-Whitney U test was used for ordinal variables such as analyte levels, since data were not normally distributed. The chi-square test was used to compare nominal variables such as diabetes mellitus. Correlations were determined by the Spearman's rho test. As we expected that preoperative PVR, duration of retinal detachment, and macular involvement were not equally distributed between both groups, we investigated the role of these clinical parameters as possible confounding variables with respect to the association between cytokine levels and the development of postoperative PVR, using multivariate logistic regression analysis. Statistical analysis of the data was performed using SPSS version 16.0 (SPSS for Windows; SPSS, Chicago, IL, USA). Differences were considered significant at *P*<0.05, with two-tailed testing.

Stepwise logistic regression analysis was used to assess the data's predictive ability in determining the occurrence of a redetachment due to postoperative PVR. Since the choice of *P*<0.05 is often too stringent in this analysis and may exclude important variables from the model, choosing a higher *P* value is highly recommended [25]. Therefore, clinical and biological variables that reached *P* values<0.15 in univariate tests were further analyzed with forward stepwise logistic regression analysis. Linear regression analysis was used to assess the data's predictive value in determining postoperative visual acuity.

## Results

### Demographics and Clinical Results

Subretinal fluid samples from 75 patients who underwent scleral buckling surgery were analyzed. Twenty one patients with primary RRD who developed postoperative PVR within 2½ months after the surgical procedure were compared with 54 patients with primary RRD who had an uncomplicated follow-up. The PVR group consisted of 6 women (29%) and 15 men (71%) with a median age of 62 years (range 43–76). The median time interval between the scleral buckling procedure and redetachment due to PVR was 37 days (range 13–80) and the median follow-up time was 21 months (range 3–80). Ten patients developed a redetachment due to postoperative PVR grade B, 10 patients due to postoperative PVR grade C, and one patient due to postoperative PVR grade D. In comparison, the RRD group included 14 women (26%) and 40 men (74%) with a median age of 61 years (range 43–79). Their median follow-up time was 6 months (range 3–80).

Potential preoperative clinical risk factors for the development of postoperative PVR were available for all 75 patients included in this study (Table 1). There were no statistically significant differences between both groups regarding these parameters, except for a worse median preoperative logMAR visual acuity in the PVR group (1.77; range 0.10–2.52) as compared to the RRD group (0.75; range 0.05–2.52) (*P* = 0.045). Although not statistically significant, median preoperative PVR grades were higher in the PVR group (grade B) than in the RRD group (grade A) (*P* = 0.218), as well as the median duration of retinal detachment (PVR, 6 days; RRD, 5 days) (*P* = 0.650) and the percentage of patients with macular involvement (PVR, 86%; RRD 64%) (*P* = 0.067).

### Interleukin and Growth Factor Levels in Subretinal Fluid

Among the interleukins analyzed, levels of IL-1α (*P* = 0.025), IL-2 (*P* = 0.044), IL-3 (*P* = 0.008), IL-6 (*P*<0.001), IL-15 (*P* = 0.013), and IL-18 (*P* = 0.008) were significantly higher in patients with primary RRD who developed a redetachment due to PVR than in patients with primary RRD and an uncomplicated follow-up. Since small nonsignificant differences in baseline characteristics between both groups may have influenced our results, we decided to correct for preoperative PVR grade, duration of retinal detachment, and macular involvement using multivariate logistic regression analysis. After correction, levels of IL-1α (*P* = 0.011), IL-2 (*P* = 0.021), IL-3 (*P* = 0.006), and IL-6 (*P* = 0.021) remained significantly elevated in the PVR group as compared to the RRD group, whereas both IL-15 (*P* = 0.392) and IL-18 (*P* = 0.281) lost their significance.

Levels of IGF-1, bFGF, NGF, and HGF were similar between both groups (*P*>0.05), whereas median VEGF levels were approximately two to three times higher in the PVR group as compared to the RRD group (*P* = 0.012). After correction for the aforementioned preoperative clinical parameters, this difference remained statistically significant (*P* = 0.049). In the current study, we found wide variations in the levels of both IL-6 and VEGF with approximately a 100-fold increase of the highest levels compared to the lowest levels in both the PVR and RRD group.

**Figure 1:**
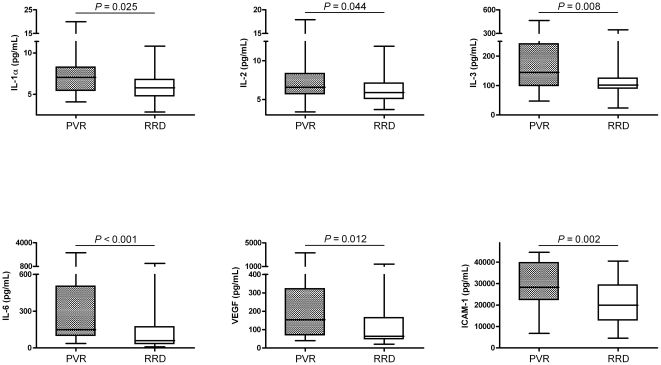
Box-and-whisker plots of significantly elevated cytokine levels in patients with primary rhegmatogenous retinal detachment. A multiplex immunoassay was used to determine 29 different cytokines in subretinal fluid samples obtained during scleral buckling surgery for rhegmatogenous retinal detachment. Patients who developed a redetachment due to postsurgical PVR within 2½ months after reattachment surgery (*n* = 21, ‘PVR’ group) were compared with controls who had an uncomplicated retinal detachment during the overall follow-up period (*n* = 54, ‘RRD’ group). Levels of IL-1α, IL-2, IL-3, IL-6, VEGF, and ICAM-1 were significantly higher (*P*<0.05) in patients who developed postoperative PVR than in patients with an uncomplicated postoperative course. After correction for possible confounding variables including preoperative PVR, duration of retinal detachment, and macular involvement, these factors remained significantly different between both groups (see text). *Box*: lower and upper quartiles; *horizontal line*: the median. Abbreviations: PVR = proliferative vitreoretinopathy; RRD = rhegmatogenous retinal detachment; IL = interleukin; VEGF = vascular endothelial growth factor; ICAM-1 = intercellular adhesion molecule-1.

IFN-γ was detected in 9 of 54 (17%) subretinal fluid samples from patients in the RRD group and in 3 of 21 (14%) samples from patients in the PVR group (*P*>0.05). There were no significant differences in the median levels of IFN-γ and TNF-α between the two groups (*P*>0.05). The median levels of the cell adhesion molecule ICAM-1 were significantly elevated in the PVR group as compared to the RRD group (*P* = 0.002), even after having corrected for differences in preoperative PVR grade, duration of retinal detachment, and macular involvement (*P* = 0.016). Levels of all cytokines are summarized in Table 2; levels of cytokines that were significantly different between both groups are illustrated in Fig. 1.

**Table 2:** Cytokine Levels and the Development of Postoperative PVR.

Cytokine (pg/mL)	RRD Group (*n* = 54)	PVR Group (*n* = 21)	Univariate testing	Stepwise Logistic Regression
IL-1α	5.8 (2.9–11)	7.0 (4.1–20)	*P* = 0.025	NS
IL-1β	6.4 (2.5–12)	6.8 (3.1–20)	NS	NS
IL-2	5.9 (3.7–12)	6.6 (3.4–18)	*P* = 0.044	NS
IL-3	101 (24–348)	144 (47–464)	*P* = 0.008	*P* = 0.001
IL-4	1.5 (0.5–2.8)	1.7 (1.0–4.5)	NS	NS
IL-5	2.3 (0.8–4.5)	2.2 (0.9–6.3)	NS	NS
IL-6	60 (8.3–1211)	149 (36–2656)	*P*<0.001	*P* = 0.047
IL-7	16 (6.2–63)	17 (6.7–53)	NS	NS
IL-9	103 (49–185)	96 (54–288)	NS	NS
IL-10	3.5 (1.7–8.4)	3.8 (2.3–8.5)	NS	NS
IL-11	23 (7.3–59)	25 (12–89)	NS	NS
IL-12p70	32 (15–58)	30 (19–78)	NS	NS
IL-13	59 (27–191)	57 (37–162)	NS	NS
IL-15	0.8 (0.1–15)	1.8 (0.3–11)	*P* = 0.013	NS
IL-17	37 (11–76)	32 (14–119)	NS	NS
IL-18	24 (5.8–262)	36 (12–144)	*P* = 0.008	NS
IL-21	3524 (2291–5481)	3421 (1981–7135)	NS	NS
IL-22	30 (6.6–81)	35 (9.4–98)	NS	NS
IL-23	133 (63–857)	135 (80–435)	NS	NS
IL-25	2349 (1253–65737)	2458 (1516–26189)	NS	NS
IL-33	14 (4.8–64)	17 (5.4–55)	NS	NS
TNF-α	6.2 (2.9–8.8)	6.6 (2.1–30)	NS	NS
IFN-γ	<0.3 (<0.3–16)	<0.3 (<0.3–18)	NS	NS
IGF-1	1548 (1086–2344)	1607 (1106–3401)	NS	NS
bFGF	1137 (715–2051)	1154 (767–2869)	NS	NS
NGF	7.0 (2.8–9.6)	7.5 (2.8–31)	NS	NS
HGF	3413 (413–9987)	3949 (971–10769)	NS	NS
VEGF	64 (21–1421)	153 (40–3308)	*P* = 0.012	NS
ICAM-1	19927 (4529–40489)	28276 (6780–44606)	*P* = 0.002	*P* = 0.010

Data are expressed as median (range) in pg/mL. Abbreviations: RRD = rhegmatogenous retinal detachment; PVR = proliferative vitreoretinopathy; IL = interleukin; NS = not significant; TNF-α = tumor necrosis factor-α; IFN-γ = interferon-γ; IGF-1 = insulin growth factor-1; bFGF = basic fibroblast growth factor; NGF = nerve growth factor; HGF = hepatocyte growth factor; VEGF = vascular endothelial growth factor; ICAM-1 = intercellular adhesion molecule-1.

To find out whether there were differences in cytokine levels between patients with different postoperative PVR grades within the PVR group, we compared patients with postoperative PVR grade B (*n* = 10) to patients with postoperative PVR grade C or higher (*n* = 11). Median IL-6 levels were significantly higher in the PVR grade B group (245 pg/mL, range 116–2656) as compared to the PVR grade C group (114 pg/mL, range 36–1398) (*P* = 0.024). No other significant differences in cytokine levels were detected between both groups. Comparison of cytokines between patients who developed postoperative PVR within 30 days (*n* = 8) and those who developed PVR after 30 days following surgery (*n* = 13) did not reveal any significant differences.

### Correlations of Biological Variables and Clinical Variables

There were moderate to strong correlations between IL-1α and both IL-2 and IL-3 (*r* = 0.83 and *r* = 0.64, respectively) (*P*<0.001) and between IL-2 and IL-3 (*r* = 0.47) (*P*<0.001). Further, we found a strong correlation between VEGF and ICAM-1 (*r* = 0.61). When comparing cytokine levels with clinical variables, we generally found low correlations (*r*<0.4) except between duration of macular detachment and ICAM-1 (*r* = 0.40) (*P*<0.01). With respect to preoperative PVR grade, we only found a very weak association with ICAM-1 levels (*r* = 0.25) (*P* = 0.030). Preoperative visual acuity correlated moderately but significantly with final visual acuity (*r* = 0.43) (*P*<0.001). Correlations between other preoperative clinical variables (i.e. duration of retinal detachment, duration of macular detachment, the extent of retinal detachment in quadrants, and the number of retinal breaks) and final visual acuity were very low (*r*<0.3), whereas patients with macular detachment at the time of primary retinal detachment surgery had worse final visual outcome (median logMAR VA 0.52, range 0–3.00) as compared to patients without involvement of the macula (median logMAR VA 0.30, range 0–0.80) (*P* = 0.013).

### Predictors of Postoperative PVR and Visual Outcome

Variables that were significantly different between the RRD group and the PVR group or were close to significance in univariate tests (*P*<0.15) were selected for stepwise logistic regression analysis. The selected variables were IL-1α, IL-2, IL-3, IL-6, IL-15, IL-18, IL-25, NGF, VEGF, ICAM-1, preoperative logMAR visual acuity and presence of macular detachment. This analysis revealed that IL-3 (*P* = 0.001), IL-6 (*P* = 0.047), ICAM-1 (*P* = 0.010), and preoperative logMAR visual acuity (*P* = 0.026) were independent predictors of postoperative PVR after primary RRD. On the other hand, linear regression analysis showed that ICAM-1 (*P* = 0.005) and preoperative logMAR visual acuity (*P* = 0.001) were predictive of final visual outcome after primary RRD repair.

## Discussion

In the current study, we found levels of IL-1α, IL-2, IL-3, IL-6, VEGF, and ICAM-1 to be significantly higher in patients who developed a redetachment due to postoperative PVR than in patients with an uncomplicated follow-up after primary RRD. Correction for possible confounding variables including preoperative PVR, duration of retinal detachment, and macular involvement was necessary due to small differences in baseline characteristics between the PVR group and the RRD group. Stepwise forward logistic regression analysis revealed that IL-3, IL-6, ICAM-1, and preoperative visual acuity were independent predictors of postoperative PVR, whereas ICAM-1 and preoperative visual acuity were predictive of final visual acuity after primary RRD repair. Samples from patients with preoperative conditions known to induce PVR were excluded from the study, since these would have yielded high cytokine levels that were directly related to that condition (e.g., uveitis). In addition, we excluded samples from patients with late PVR development, considering it would be more likely that in patients who have PVR developed after 3 months, a new event caused the recurrence rather than the stimulus that was of our interest [26]. Hence, our findings indicate that there is an association between certain cytokines (IL-1α, IL-2, IL-3, IL-6, VEGF, and ICAM-1) and the development of postsurgical PVR after primary RRD repair. Whether an unknown clinical event may have triggered the exaggerated cytokine response in the PVR group or whether differences in the genetic control of this cytokine response are involved remains to be clarified.

Animal studies have shown an upregulation of proinflammatory genes already very soon (hours to days) after the induction of retinal detachment together with a time dependency of certain cytokines [16], [17]. These animal studies are certainly far more well-controlled than our clinical study and a time dependency could not be analyzed due to insufficient time points. Nevertheless, we demonstrated that sampling after the onset of primary RRD but before the development of postoperative PVR may provide clues as to which biological events initiate the formation of PVR membranes, and may therefore provide a means for therapeutic control before postoperative PVR has been established. In the scope of developing a preventive strategy, a study by Bali et al [27] showed that a single preoperative subconjunctival injection of dexamethasone prior to scleral buckling retinal detachment surgery resulted in a significant decrease in laser flare measurements at one week postoperatively, reflecting less blood-retina barrier breakdown. Whether steroid priming via these injections leads to a lower incidence of postoperative PVR has not yet been investigated.

Multiplex bead-based immunoassay allowed us to detect a large number of cytokines simultaneously, with comparable performance in sensitivity, accuracy, and reproducibility to enzyme-linked immunosorbent assays (ELISAs) performed in previous studies [28]. With the use of ELISAs, earlier studies could only focus on a few cytokines and mostly studied patients with already established PVR following earlier reattachment surgery. Moreover, most studies have dealt with vitreous samples, whereas our study addressed subretinal fluid specimens. To discover early biological alterations that may lead to PVR development, analysis of subretinal fluid may be more appropriate because of its close proximity to the RPE cell layer after initial retinal detachment.

The source of the cytokines in our subretinal fluid specimens remains speculative although it is most likely that they are simultaneously produced by resident ocular cells and infiltrating inflammatory cells. Even though the blood-retina barrier may be broken down, the levels of plasma cytokines do not reach high enough levels to significantly contribute to the cytokine milieu in the subretinal space after retinal detachment [18], [19], [29].

A large number of ocular fluid cytokines has been investigated previously in RRD patients with or without PVR, including IL-1, IL-6, TNF-α, IFN-γ, VEGF, FGF, HGF, and ICAM-1 (Table S1) [8]–[12], [18]–[21], [29]–[33]. Of this group of cytokines, we confirmed earlier findings showing elevated levels of IL-1α, IL-6, VEGF, and ICAM-1 in our specimens. Comparison of the levels reported in these studies is however difficult due to significant differences in sampling time, sampling size, sampling specimen, laboratory methods, and baseline characteristics of the study population. A correlation between increased levels of IL-2 and IL-3 and the development of postoperative PVR as we describe in the current study has not yet been reported earlier.

The proinflammatory cytokines IL-1α and IL-1β have similar biological properties and have been implicated in the pathogenesis of PVR [8], [9]. Since IL-1 induces RPE cell migration [34] and its intravitreal injection leads to breakdown of the blood-ocular barrier [35], IL-1 has been suggested to be an important candidate in the activation processes that ultimately lead to PVR development. Although our findings showed that IL-1α levels were significantly elevated in the PVR group, the difference with the control group was only small. The same holds for IL-2 with median levels of less than 7 pg/mL. On the other hand, many cytokines have redundant properties and even small elevations in a number of cytokines may lead to synergistic effects.

IL-6 reached very high levels in some patients and median levels were approximately 2.5 times higher in the PVR group as compared to the RRD group. IL-6 is a pleiotropic cytokine with a wide range of activities in inflammation and immune reactions [36]. Various groups have reported IL-6 gene expression and secretion by cytokine-stimulated human RPE cells [37], [38], whereas IL-6 may also be produced by several inflammatory cells invading the subretinal space after RRD due to chemotactic signalling [39]. In addition, IL-6 itself was found to induce chemokines and to amplify the recruitment of leukocytes in an animal model [40]. Consistent with these data, we reported the correlation between IL-6 and a wide range of chemokines in subretinal fluid samples from patients with retinal detachment [22]. IL-6, in contrast, may also serve as a photoreceptor neuroprotectant, as was shown in an experimental model of retinal detachment [41]. Results of our current study were in line with those of Kon et al [8] and El-Ghrably et al [9], suggesting a role for IL-6 in the early alterations that may induce postoperative PVR (Table S1). Interestingly, we found significantly lower IL-6 levels in patients with higher postoperative PVR grades, for which we have no sound explanation.

Similar to IL-6, we found median VEGF levels to be two to three times elevated in subretinal fluid samples of patients with PVR. Since PVR is characterized by the formation of mainly avascular membranes, it was hypothesized that VEGF may have functions other than inducing angiogenesis [4]. This hypothesis was supported by growing evidence of a possible interaction of VEGF with non-endothelial cells in the eye. A previous study has shown that RPE cells in situ, as well as RPE cells in epiretinal membranes and in culture, express VEGF receptors [4]. In the majority of these membranes, VEGF and its receptors were co-localized, indicating that an autocrine and/or paracrine mechanism may exist. Other studies identified VEGF receptors on other non-endothelial cells in the eye such as on adult photoreceptor cells and Müller cells, implicating a role for VEGF in the maintenance of the adult neural retina by supporting cell survival of these retinal cells [42]. Interestingly, VEGF has also been shown to induce monocyte activation manifested by the induction of monocyte chemotaxis [7]. Thus, VEGF probably exerts pleiotropic effects on diverse cell types such as RPE cells and monocytes, which play major roles in the development of PVR.

The presence of ICAM-1 in epiretinal membranes and vitreous of patients with PVR has been demonstrated earlier in a few studies [43], [44]. In addition, ICAM-1 expression has been found on cultured human RPE cells and was upregulated after stimulation with IL-1, TNF-α, or IFN-γ [45]. ICAM-1 has an important role in regulating leukocyte migration into sites of inflammation, thereby enhancing cytokine-mediated inflammatory reactions after retinal detachment. Limb and Chignell [10] showed that high levels of ICAM-1 may constitute an additional risk factor to known clinical risk factors for the development of PVR. Our study confirmed these results, and we showed that ICAM-1 is not only an independent predictor of postoperative PVR, but also of visual outcome after reattachment surgery.

The use of multiplex immunoassays entails research that is basically hypothesis-generating. We identified IL-2 and IL-3 as novel candidates in the activation of PVR development. Elevated concentrations of these cytokines in our samples suggest their involvement in the initial stages of PVR pathogenesis. IL-3, which was shown to be the best predictor of postoperative PVR in our study population, exerts proinflammatory actions by the activation of hematopoietic cells of various lineages including macrophages [46], and is mainly synthesized by T-lymphocytes [47]. Together with studies showing their presence in ocular fluids and epiretinal membranes from patients with PVR [48], [49], our results underscore a possible role for T lymphocytes in the pathogenesis of PVR. Since microglial cells have also been shown to express IL-3 [50], its cellular source and function after retinal detachment remain unknown.

So far, the use of intravitreal pharmacologic agents has not been proven to be efficacious in the prevention or treatment of PVR and most efforts have been dedicated to modulate the clinical risk factors [13], [14]. Despite these efforts PVR is still the primary cause of failure of retinal detachment surgery and a frequent cause of legal blindness in developed countries. Therefore, more insight in its pathogenesis is needed to develop further prevention strategies. The identification of laboratory predictors of PVR (IL-3, IL-6, ICAM-1) may help us to treat only those patients at greatest risk. On the other hand, the identification of preoperative visual acuity as an independent predictor of postoperative PVR should be viewed with caution since it is merely a result of statistical analysis and is most likely due to a higher proportion of patients with macular involvement in the PVR group. Future prospective studies are necessary to test the validity of our study. Since we only assessed cytokine content in subretinal fluid samples from patients who underwent scleral buckling surgery for primary RRD, predictors of postoperative PVR derived from our study results may not be applicable to other patient populations (e.g., patients who undergo a vitrectomy). The present study did not include known PVR associated factors such as platelet-derived growth factor (PDGF) or transforming growth factor (TGF)-β [30], [51]. The PDGF assay has not yet been developed by the Luminex Core Facility, whereas the TGF-β assay needs a prior acid activation to convert the latent into the activated form, which prevents its simultaneous detection with other cytokines in the multiplex bead immunoassay.

In summary, our findings show that several cytokines are upregulated after the onset of RRD in patients in whom postoperative PVR develops after primary RRD repair. An exaggerated inflammatory response in this subset of patients may thus be the underlying phenomenon that ultimately leads to the formation of PVR membranes. Moreover, we report that IL-3, IL-6, ICAM-1, and preoperative visual acuity were independent predictors of PVR and may be useful in identifying those patients at greatest risk. Importantly, elevated levels of IL-3 may shed new light on PVR pathogenesis and may suggest T cell and/or microglial cell involvement in the early events of PVR development.

## Supporting Information

Table S1 Summary of cytokine levels in RRD patients with and without PVR.
(DOC)

## References

[1] D'Amico DJ (2008). Clinical practice. Primary retinal detachment.. N Engl J Med.

[2] Pastor JC, de la Rúa ER, Martín F (2002). Proliferative vitreoretinopathy: risk factors and pathobiology.. Prog Retin Eye Res.

[3] Fredj-Reygrobellet D, Baudouin C, Nègre F, Caruelle JP, Gastaud P (1991). Acidic FGF and other growth factors in preretinal membranes from patients with diabetic retinopathy and proliferative vitreoretinopathy.. Ophthalmic Res.

[4] Chen YS, Hackett SF, Schoenfeld CL, Vinores MA, Vinores SA (1997). Localisation of vascular endothelial growth factor and its receptors to cells of vascular and avascular epiretinal membranes.. Br J Ophthalmol.

[5] Spraul CW, Kaven C, Lang GK, Lang GE (2004). Effect of growth factors on bovine retinal pigment epithelial cell migration and proliferation.. Ophthalmic Res.

[6] Grant MB, Guay C, Marsh R (1990). Insulin-like growth factor I stimulates proliferation, migration, and plasminogen activator release by human retinal pigment epithelial cells.. Curr Eye Res.

[7] Clauss M, Gerlach M, Gerlach H, Brett J, Wang F (1990). Vascular permeability factor: a tumor-derived polypeptide that induces endothelial cell and monocyte procoagulant activity, and promotes monocyte migration.. J Exp Med.

[8] Kon CH, Occleston NL, Aylward GW, Khaw PT (1999). Expression of vitreous cytokines in proliferative vitreoretinopathy: a prospective study.. Invest Ophthalmol Vis Sci.

[9] El-Ghrably IA, Dua HS, Orr GM, Fischer D, Tighe PJ (2001). Intravitreal invading cells contribute to vitreal cytokine milieu in proliferative vitreoretinopathy.. Br J Ophthalmol.

[10] Limb GA, Chignell AH (1999). Vitreous levels of intercellular adhesion molecule 1 (ICAM-1) as a risk indicator of proliferative vitreoretinopathy.. Br J Ophthalmol.

[11] Kauffmann DJH, Van Meurs JC, Mertens DAE, Peperkamp E, Master C (1994). Cytokines in vitreous humor: interleukin-6 is elevated in proliferative vitreoretinopathy.. Invest Ophthalmol Vis Sci.

[12] La Heij EC, Van de Waarenburg MP, Blaauwgeers HG, Kessels AGH, Liem ATA (2002). Basic fibroblast growth factor, glutamine synthetase, and interleukin-6 in vitreous fluid from eyes with retinal detachment complicated by proliferative vitreoretinopathy.. Am J Ophthalmol.

[13] Wickham L, Bunce C, Wong D, McGurn D, Charteris DG (2007). Randomized controlled trial of combined 5-fluorouracil and low-molecular-weight heparin in the management of unselected rhegmatogenous retinal detachments undergoing primary vitrectomy.. Ophthalmology.

[14] Ahmadieh H, Feghhi M, Tabatabaei H, Shoeibi N, Ramezani A (2008). Triamcinolone acetonide in silicone-filled eyes as adjunctive treatment for proliferative vitreoretinopathy: a randomized clinical trial.. Ophthalmology.

[15] Wiedemann P, Hilgers RD, Bauer P, Heimann K (1998). Adjunctive daunorubicin in the treatment of proliferative vitreoretinopathy: results of a multicenter clinical trial. Daunomycin Study Group.. Am J Ophthalmol.

[16] Hollborn M, Francke M, Iandiev I, Bühner E, Foja C (2008). Early activation of inflammation- and immune response-related genes after experimental detachment of the porcine retina.. Invest Ophthalmol Vis Sci.

[17] Nakazawa T, Matsubara A, Noda K, Hisatomi T, She H (2006). Characterization of cytokine responses to retinal detachment in rats.. Mol Vis.

[18] Canataroglu H, Varinli I, Ozcan AA, Canataroglu A, Doran F (2005). Interleukin (IL)-6, interleukin (IL)-8 levels and cellular composition of the vitreous humor in proliferative diabetic retinopathy, proliferative vitreoretinopathy, and traumatic proliferative vitreoretinopathy.. Ocul Immunol Inflamm.

[19] Su CY, Chen MT, Wu WS, Wu WC (2000). Concentration of vascular endothelial growth factor in the subretinal fluid of retinal detachment.. J Ocul Pharmacol Ther.

[20] Mitamura Y, Takeuchi S, Matsuda A, Tagawa Y, Mizue Y (2000). Hepatocyte growth factor levels in the vitreous of patients with proliferative vitreoretinopathy.. Am J Ophthalmol.

[21] Ogata N, Nishikawa M, Nishimura T, Mitsuma Y, Matsumura M (2002). Inverse levels of pigment epithelium-derived factor and vascular endothelial growth factor in the vitreous of eyes with rhegmatogenous retinal detachment and proliferative vitreoretinopathy.. Am J Ophthalmol.

[22] Ricker LJAG, Kijlstra A, De Jager W, Liem ATA, Hendrikse H (2010). Chemokine levels in subretinal fluid obtained during scleral buckling surgery after rhegmatogenous retinal detachment.. Invest Ophthalmol Vis Sci.

[23] de Jager W, Bourcier K, Rijkers GT, Prakken BJ, Seyfert-Margolis V (2009). Prerequisites for cytokine measurements in clinical trials with multiplex immunoassays.. BMC Immunol.

[24] Retina Society Terminology Committee (1983). The classification of retinal detachment with proliferative vitreoretinopathy.. Ophthalmology.

[25] Hosmer DW, Lemeshow S, Shewhart WA, Wilks SS (1989). Model-building strategies and methods for logistic regression.. Applied logistic regression.

[26] Mietz H, Heimann K (1995). Onset and recurrence of proliferative vitreoretinopathy in various vitreoretinal diseases.. Br J Ophthalmol.

[27] Bali E, Feron EJ, Peperkamp E, Veckeneer M, Mulder PG (2010). The effect of a preoperative subconjunctival injection of dexamethasone on blood-retinal barrier breakdown following scleral buckling retinal detachment surgery: a prospective randomized placebo-controlled double blind clinical trial.. Graefes Arch Clin Exp Ophthalmol.

[28] de Jager W, Te Velthuis H, Prakken BJ, Kuis W, Rijkers GT (2003). Simultaneous detection of 15 human cytokines in a single sample of stimulated peripheral blood mononuclear cells.. Clin Diagn Lab Immunol.

[29] Yoshimura T, Sonoda KH, Sugahara M, Mochizuki Y, Enaida H (2009). Comprehensive analysis of inflammatory immune mediators in vitreoretinal diseases.. PLoS ONE.

[30] Dieudonné SC, La Heij EC, Diederen R, Kessels AGH, Liem ATA (2004). High TGF-β2 levels during primary retinal detachment may protect against proliferative vitreoretinopathy.. Invest Ophthalmol Vis Sci.

[31] Banerjee S, Savant V, Scott RAH, Curnow SJ, Wallace GR (2007). Multiplex bead analysis of vitreous humor of patients with vitreoretinal disorders.. Invest Ophthalmol Vis Sci.

[32] Rasier R, Gormus U, Artunay O, Yuzbasioglu E, Oncel M (2010). Vitreous levels of VEGF, IL-8, and TNF-alpha in retinal detachment.. Curr Eye Res.

[33] Dieudonné SC, La Heij EC, Diederen RMH, Kessels AGH, Liem ATA (2007). Balance of vascular endothelial growth factor and pigment epithelial growth factor prior to development of proliferative vitreoretinopathy.. Ophthalmic Res.

[34] Kirchhof B, Kirchhof E, Ryan SJ, Dixon JF, Barton BE (1989). Macrophage modulation of retinal pigment epithelial cell migration and proliferation.. Graefes Arch Clin Exp Ophthalmol.

[35] Ferrick MR, Thurau SR, Oppenheim MH, Herbort CP, Ni M (1991). Ocular inflammation stimulated by intravitreal interleukin-8 and interleukin-1.. Invest Ophthalmol Vis Sci.

[36] Kishimoto T (2006). Interleukin-6: discovery of a pleiotropic cytokine.. Arthritis Res Ther.

[37] Kuppner MC, McKillop-Smith S, Forrester JV (1995). TGF-beta and IL-1 beta act in synergy to enhance IL-6 and IL-8 mRNA levels and IL-6 production by human retinal pigment epithelial cells.. Immunology.

[38] Holtkamp GM, Van Rossem M, de Vos AF, Willekens B, Peek R (1998). Polarized secretion of IL-6 and IL-8 by human retinal pigment epithelial cells.. Clin Exp Immunol.

[39] de Vos AF, Hoekzema R, Kijlstra A (1992). Cytokines and uveitis, a review.. Curr Eye Res.

[40] Romano M, Sironi M, Toniatti C, Polentarutti N, Fruscella P (1997). Role of IL-6 and its soluble receptor in induction of chemokines and leukocyte recruitment.. Immunity.

[41] Chong DY, Boehlke CS, Zheng QD, Zhang L, Han Y (2008). Interleukin-6 as a photoreceptor neuroprotectant in an experimental model of retinal detachment.. Invest Ophthalmol Vis Sci.

[42] Saint-Geniez M, Maharaj AS, Walshe TE, Tucker BA, Sekiyama E (2008). Endogenous VEGF is required for visual function: evidence for a survival role on Müller cells and photoreceptors.. PLoS ONE.

[43] Limb GA, Franks WA, Munasinghe KR, Chignell AH, Dumonde DC (1993). Proliferative vitreoretinopathy: an examination of the involvement of lymphocytes, adhesion molecules and HLA-DR antigens.. Graefes Arch Clin Exp Ophthalmol.

[44] Esser P, Bresgen M, Fischbach R, Heimann K, Wiedemann P (1995). Intercellular adhesion molecule-1 levels in plasma and vitreous from patients with vitreoretinal disorders.. Ger J Ophthalmol.

[45] Elner SG, Elner VM, Pavilack MA, Todd RF, Mayo-Bond L (1992). Modulation and function of intercellular adhesion molecule-1 (CD54) on human retinal pigment epithelial cells.. Lab Invest.

[46] Frendl G, Beller DI (1990). Regulation of macrophage activation by IL-3. I. IL-3 functions as a macrophage-activating factor with unique properties, inducing Ia and lymphocyte function-associated antigen-1 but not cytotoxicity.. J Immunol.

[47] Lindemann A, Mertelsmann R (1993). Interleukin-3: structure and function.. Cancer Invest.

[48] Charteris DG, Hiscott P, Robey HL, Gregor ZJ, Lightman SL (1993). Inflammatory cells in proliferative vitreoretinopathy subretinal membranes.. Ophthalmology.

[49] Baudouin C, Hofman P, Brignole F, Bayle J, Loubière R (1991). Immunocytology of cellular components in vitreous and subretinal fluid from patients with proliferative vitreoretinopathy.. Ophthalmologica.

[50] Gebicke-Haerter PJ, Appel K, Taylor GD, Schobert A, Rich IN (1994). Rat microglial interleukin-3.. J Neuroimmunol.

[51] Lei H, Rheaume MA, Kazlauskas A (2010). Recent developments in our understanding of how platelet-derived growth factor (PDGF) and its receptors contribute to proliferative vitreoretinopathy.. Exp Eye Res.

