# Supplementary material for: Interleukin and Growth Factor Levels in Subretinal Fluid in Rhegmatogenous Retinal Detachment: A Case-Control Study
Source: PLoS One. 2011 Apr 27;6(4):e19141. doi: 10.1371/journal.pone.0019141 (PMC3083411; doi:10.1371/journal.pone.0019141)
Supplement: Table S1 — Summary of cytokine levels in RRD patients with and without PVR. (DOC) [file pone.0019141.s001.doc]

**Table S1 Summary of cytokine levels in RRD patients with and without PVR**

| **Study (Sample)** | **Patient Group (n)** | **IL-1α** | **IL-1β** | **IL-6** | **TNF-α** | **IFN-γ** | **VEGF** | **FGF** | **HGF** | **ICAM-1** |
| --- | --- | --- | --- | --- | --- | --- | --- | --- | --- | --- |
| Current study (SF) | PVR (n = 21) | 7.0 (4.1-20)* | 6.8 (3.1-20) | 149 (36-2656)* | 6.6 (2.1-30) | <0.3 (<0.3-18) | 153 (40-3308)* | 1154 (767-2869) | 3949 (971-10769) | 28276 (6780-44606)* |
|  | RRD (n = 54) | 5.8 (2.9-11) | 6.4 (2.5-12) | 60 (8.3-1211) | 6.2 (2.9-8.8) | <0.3 (<0.3-16) | 64 (21-1421) | 1137 (715-2051) | 3413 (413-9987) | 19927 (4529-40489) |
| Reference 8 (VF) | PVR (n = 40) |  | 4.0 (3.1-5.0) | 319 (226-411)* |  |  |  | 83 (47-120)* |  |  |
|  | RRD (n = 96) |  | 4.2 (3.6-4.8) | 163 (128-198) |  |  |  | 38 (24-53) |  |  |
| Reference 9 (VF) | PVR (n = 15) | 18 | 0.82* | 191* | ND | 201 |  |  |  |  |
|  | RRD (n = 8) | 6.3 | 0.34 | 42 | ND | 27 |  |  |  |  |
| Reference 10 (VF) | ↑RRD (n = 36) |  |  |  |  |  |  |  |  | 6100-97700* |
|  | ↓RRD (n = 31) |  |  |  |  |  |  |  |  | 4800-17700 |
| Reference 11 (VF) | PVR (n = 14) |  | ND | 92 (18) | ND |  |  |  |  |  |
| Reference 12 (VF) | PVR (n = 53) |  |  | 396 |  |  |  | 124 |  |  |
| Reference 18 (VF) | PVR (n = 10) |  |  | 100 (139) |  |  |  |  |  |  |
| Reference 19 (SF) | PVR (n = 8) |  |  |  |  |  | 901 (385)* |  |  |  |
|  | RRD (n = 31) |  |  |  |  |  | 355 (373) |  |  |  |
| Reference 20 (VF) | PVR (n = 23) |  |  |  |  |  |  |  | 3940 (2290)* |  |
|  | RRD (n = 22) |  |  |  |  |  |  |  | 2020 (840) |  |
| Reference 21 (VF) | PVR (n = 6) |  |  |  |  |  | 168 (139)* |  |  |  |
|  | RRD (n = 26) |  |  |  |  |  | 11 (11) |  |  |  |
| Reference 29 (VF) | RRD (n = 63) |  | ND | 702 (<30-15381) | ND | ND | 78 (<150-712) | ND |  |  |
| Reference 30 (SF) | PVR (n = 30) |  |  |  |  |  |  |  | 10000 (5900) |  |
|  | RRD (n = 114) |  |  |  |  |  |  |  | 10800 (9500) |  |
| Reference 31 (VF) | PVR (n = 8) |  |  | 975 (0-14182) | 12 (0-31) | 5 (0-40) | 975 (0-14182) | 28 (0-255) |  |  |
| Reference 32 (VF) | RRD (n = 22) |  |  |  | 58 (5.2) |  | 782 (390) |  |  |  |
| Reference 33 (SF) | PVR (n = 17) |  |  |  |  |  | 20 (10-6410) |  |  |  |
|  | RRD (n = 46) |  |  |  |  |  | 80 (10-8470) |  |  |  |

Data are expressed as mean (standard deviation), median (range), median only, or range only in pg/mL.^*^ = significantly higher levels in PVR group compared to RRD group; ↑RRD = high risk RRD; ↓RRD = low risk RRD. Abbreviations: IL = interleukin; TNF-α = tumor necrosis factor-α; IFN-γ = interferon-γ; VEGF = vascular endothelial growth factor; FGF = fibroblast growth factor; HGF = hepatocyte growth factor; ICAM-1 = intercellular adhesion molecule-1; SF = subretinal fluid; PVR = proliferative vitreoretinopathy; RRD = rhegmatogenous retinal detachment; VF = vitreous fluid; ND = not detected.
